# Myofibroblast expression in airways and alveoli is affected by smoking and COPD

**DOI:** 10.1186/1465-9921-14-84

**Published:** 2013-08-11

**Authors:** Henna M Karvonen, Siri T Lehtonen, Terttu Harju, Raija T Sormunen, Elisa Lappi-Blanco, Johanna M Mäkinen, Kirsi Laitakari, Shirley Johnson, Riitta L Kaarteenaho

**Affiliations:** 1 Department of Internal Medicine / Respiratory Research Unit, Institute of Clinical Medicine, University of Oulu, Oulu, Finland; 2 Respiratory Research Unit and Medical Research Center Oulu, Oulu University Hospital, Oulu, Finland; 3 Department of Anatomy and Cell Biology, Institute of Biomedicine, University of Oulu, Oulu, Finland; 4 Biocenter Oulu, University of Oulu, Oulu, Finland; 5 Department of Pathology, Institute of Diagnostics, University of Oulu, Oulu, Finland; 6 Department of Pathology, Oulu University Hospital, Oulu, Finland; 7 Unit of Medicine and Clinical Research, Pulmonary Division, University of Eastern Finland, Kuopio, Finland; 8 Center for Medicine and Clinical Research, Division of Respiratory Medicine, Kuopio University Hospital, PO Box 100 70029, Kuopio, Finland

**Keywords:** Bronchiolus, Bronchus, Emphysema, Electron microscopy, Fibroblast, Western blotting

## Abstract

**Background:**

Chronic obstructive pulmonary disease (COPD) is characterized by structural changes in alveoli and airways. Our aim was to analyse the numbers of alpha-smooth muscle actin (α-SMA) positive cells, as a marker of myofibroblasts, in different lung compartments in non-smokers and smokers with normal lung function or COPD.

**Methods:**

α-SMA, tenascin-C (Tn-C) and EDA-fibronectin in alveolar level and airways were assayed by immunohistochemistry and quantified by image analysis. Immunohistochemical findings were correlated with clinical data. α-SMA protein was also analysed by Western blotting from fibroblastic cells cultured from peripheral lung of non-smokers, smokers without COPD and smokers with COPD.

**Results:**

In many cases, the endings of the detached alveolar walls were widened, the structures of which were named as widened alveolar tips. Widened alveolar tips contained α-SMA positive cells, which were obviously myofibroblasts. There were less alveolar tips containing positive cells for α-SMA in alveoli and α-SMA positive cells in bronchioles in smokers and in COPD compared to non-smokers. The quantity of α-SMA positive cells was increased in bronchi in COPD. Tn-C was elevated in bronchi in COPD and smokers’ lung. The α-SMA protein level was 1.43-fold higher in stromal cells cultured from non-smokers than in those of smokers.

**Conclusions:**

Myofibroblasts are localized variably in normal and diseased lung. This indicates that they have roles in both regeneration of lung and pathogenesis of COPD. The widened alveolar tips, these newly characterized histological structures, seemed to be the source of myofibroblasts at the alveolar level.

## Background

Chronic obstructive pulmonary disease (COPD) is characterized by the destruction of alveoli and fibrosis of airway walls, which lead to the enlargement of air spaces i.e. emphysema, obstruction of airways and decline in lung function. The subepithelial fibrosis encountered in airways is caused by a deposition of extracellular matrix (ECM) proteins like tenascin-C (Tn-C), collagens, fibronectin (Fn) and proteoglycans [1-5]. Emphysema has been proposed to be a deficiency of alveolar regeneration. While this regenerative process is known to occur in fetal lung it can also take place in normal adult lung [6]. Alveolar mesenchymal cells may have a role in regenerative multiplication after lung tissue injury [7].

The elevated expression of ECM proteins has been shown to be associated to increased number of myofibroblasts, which are alpha-smooth muscle actin (α-SMA) positive, spindle-shaped mesenchymal cells. Myofibroblasts display a specific electron microscopic ultrastructure i.e. cell surface structure, fibronexus (FNX) that is composed of intracellular α-SMA filaments and extracellular Fn bundles, dilated rough endoplasmic reticulum (rER) and adherens-type junctions [8-11]. Myofibroblasts may be the effector cells in fibroproliferative diseases such as idiopathic pulmonary fibrosis (IPF) and COPD as well as the stromal reaction occurring in lung cancer [3,12-15]. However, the role of myofibroblasts in peripheral lung of smokers and in COPD has not yet been fully investigated, since most of the previous studies have focused on either large airways or other type of lung diseases.

Tn-C is the ECM glycoprotein which participates in the remodeling of the airways in COPD [3,5]. Tn-C also regulates cancer invasiveness by promoting epithelial-mesenchymal transition [16]. Tn-C is highly expressed during human lung development but can also be detected in many lung diseases such as asthma, IPF, asbestosis, respiratory distress syndrome (RDS), bronchopulmonary dysplasia (BPD) and granulomatous lung diseases associating with the increase of α-SMA positive cells, obvious myofibroblasts [12,17-23]. Fibroblasts from IPF-patients exhibited higher levels of extra type III domain A – fibronectin (EDA-Fn) and α-SMA when compared to normal lung fibroblasts [24].

We hypothesized that the number of α-SMA positive cells would be increased in different lung compartments i.e. alveoli, bronchioles and bronchi in COPD when compared to that of normal lung, and also that the level of α-SMA expression may be associated with those of Tn-C and EDA-Fn. The expression levels of α-SMA, Tn-C and EDA-Fn were analysed by immunohistochemistry and quantified by using image analysis. The immunohistochemical findings were correlated with the clinical data. The ultrastructure and α-SMA protein level of cells cultured from peripheral lung were studied by transmission electron microscope (TEM) and Western analysis.

## Materials and methods

### Study subjects

Lung tissue specimens from 101 patients including 24 females and 77 males (11 non-smokers including 9 life-long non-smokers and 2 ex-smokers with less than 10 pack-years, 30 current smokers with normal lung function, 9 ex-smokers with normal lung function, 26 current smokers with COPD and 20 ex-smokers with COPD) operated on for lung cancer during 1998–2007 were identified from the archive of the Department of Pathology, Oulu University Hospital. The clinical data of the patients were evaluated (Table 1). The patients were taking their daily medication i.e. 21.8% (n = 22) of the patients had inhaled corticosteroids (3 non-smokers, 2 smokers with normal lung function, 14 smokers with COPD stage II and 3 smokers with COPD stage III). According to the clinical and lung function data, the patients were divided as non-smokers, smokers with normal lung function and smokers with COPD. The definition of COPD was based on the lung function: FEV_1_/FVC < 0.70 and the classification of severity was made using the GOLD criteria: FEV_1_ ≥ 80% predicted (stage I), 50% ≤ FEV_1_ < 80% (stage II), 30% ≤ FEV_1_ < 50% (stage III) and FEV_1_ < 30% (stage IV).

**Table 1:** Characteristics of patients whose specimens were evaluated by immunohistochemistry

	**Non-smokers (n=11)**	**Smokers with normal lung function (n=41)**	**Smokers with COPD GOLD stage I (n=6)**	**Smokers with COPD GOLD stage II (n=39)**	**Smokers with COPD GOLD stage III (n=4)**	**p-value**
Age (y)	68 (7)	64 (8)	70 (9)	68 (8)	68 (10)	0.174
Sex m:f	2:9	29:12	5:1	37:2	4:0	
Pack-years	0.6 (1.3)^*^	38 (15)	40 (14)	42 (14)	42 (12)	< 0.001
FEV_1_ (L) postbd	2.4 (0.7)	2.65 (0.73)	2.82 (0.26)	2.14 (0.40)	1.49 (0.17)^¤^	< 0.001
FEV_1_% pred	94 (18)^#^	82 (16)	91 (5)	64 (8)	43 (3)	< 0.001
FEV_1_/FVC	0.80 (0.08)^$^	0.79 (0.06)	0.67 (0.007)	0.59 (0.06)	0.54 (0.05)	< 0.001
DL_CO_ (mmol/min/mmHg)	2.7 (2.1)	3.2 (2.5)	2.0 (1.9)	3.8 (2.4)	3.1 (2.7)	0.831
DL_CO_% pred	95 (17)^*^	73 (18)	65 (6)	66 (16)	67 (17)	< 0.001

Mean (SD), *The mean difference between non-smokers and smokers is significant at the 0.05 level, Dunnett t-test, ¤The mean difference between non-smokers and smokers with COPD stage III is significant at the 0.05 level, Dunnett t-test, # The mean difference between non-smokers and non-obstructed smokers or smokers with COPD stage II or stage III is significant at the 0.05 level, Dunnett t-test, $The mean difference between non-smokers and smokers with COPD is significant at the 0.05 level, Dunnett t-test , *GOLD* Global Initiative for Chronic Obstructive Lung Disease, *FEV*_*1*_ Forced expiratory volume in one second, *FVC* Forced vital capacity, *DL*_*co*_ Diffusing capacity of carbon monoxide, *postbd* Post-bronchodilator value.

### **Ethical considerations**

The study had the approval from the Ethical Committee of Northern Ostrobothnia Hospital District in Oulu (statements 64/2001, amendment 68/2005, 2/2008), and from National Supervisory Authority for Welfare and Health (former National Authority of Medicolegal Affairs, reg. nr. 7323/05.01.00.06/2009 and 863/04/047/08). For the retrospective immunohistochemical material, an informed consent permission has been given by the National Supervisory Authority for Welfare and Health, which is the national licensing authority. The study material for experiments conducted on cell lines was collected prospectively, when the patients were interviewed before the operation and samples were collected only if written consent was given.

### Immunohistochemistry

The immunohistochemical analyses of each patient were performed in two tissue slides, one from peripheral lung including small airways and alveolar level and other from large airways. Staining method and primary antibodies are represented in Additional file 1. Immunohistochemical stainings were performed in serial tissue sections.

### Image analysis

Image analyses were performed by light microscope (Leitz Wetzlar Aristoplan, Germany equipped with Canon 16 mm 1:1.4 CI-TVLENS objective, QImaging Micro Publisher 5.0 RTV digital camera and MCID™ Core Image Analysis System software). The immunohistochemical findings were studied in relation to the clinical data of the patients.

### Analyses of α-SMA, Tn-C and EDA-Fn in alveoli

The numbers of the widened alveolar tips, which were positive either for α-SMA, Tn-C or EDA-Fn, were counted one at a time in the whole area of the tissue slide. The area of lung tissue in one slide of each case was measured by image analysis. The total number of the widened alveolar tips positive for each staining was then standardized to the defined area of the peripheral lung specimen (cm^2^). Distal airspaces have been previously classified based on morphological studies and defined as a) alveoli which are hexagonal structures limited by continuous walls i.e. interalveolar septa, and b) alveolar ducts which are airway spaces limited by free tips of interalveolar septa [25,26]. In the present study, the widened alveolar tips were defined as widened endings of free interalveolar septa.

### Analyses of α-SMA, Tn-C and EDA-Fn in small airways

The bronchioles from peripheral lung tissue were recognized by a lack of cartilage. The total number of small airways i.e. bronchioles was counted in the whole area of the peripheral tissue slide. The α-SMA positivity was defined by categorizing each bronchiole into three groups (Additional file 2: Figure S1 A-C). The numbers of α-SMA positive spindle-shaped cells between epithelium and smooth muscle cell layer i.e. in the subepithelial area over the whole of bronchiolar ring were counted. The α-SMA positive cells were categorized into three groups as follows 0 (negative), 1 (< 5 positive cells) or 2 (≥ 5 positive cells) and expressed as a weighted mean in the results. Weighted mean = (0*n_1_ + 1*n_2_ + 2*n_3_) / n_total_, in which n_1_, n_2_ and n_3_ are numbers of bronchioles in each group and n_total_ = n_1_ + n_2_ + n_3_. Tn-C and EDA-Fn stainings were negative in all cases, and thus no image analysis could be performed.

### Analyses of α-SMA, Tn-C and EDA-Fn in large airways

One bronchus of each central lung specimen was analyzed. The α-SMA positive area (mm^2^) of spindle-shaped cells in the subepithelial area between epithelium and smooth muscle cell layer was manually delineated, and normalized to the length of bronchial epithelium (mm) measured at the basal side of basement membrane (BM). Smooth muscle cells and vessels were not counted when they located outside the areas of spindle-shaped myofibroblasts whereas the structures inside were included (Additional file 2: Figure S1 D). An example image of the α-SMA positive area is shown in Additional file 2: Figure S1 E.

Tn-C expression was analysed by a semi-quantitative method as previously described [3] showing positivity a) in basal cells and BM, b) in basal cells plus BM plus stroma underneath BM, and c) in above-mentioned areas plus widely in the stroma. Typical example images of different expression categories are shown in Additional file 2: Figure S1 F-H. The data were independently evaluated by two investigators (kappa coefficient = 0.776) [27]. EDA-Fn stainings were negative in all cases, and thus no image analysis could be performed.

### In vitro experiments conducted on cell lines cultured from peripheral lung tissue

The peripheral lung tissues (5 life-long non-smokers, 9 smokers without COPD and 8 patients with mild or moderate COPD) from lung resections were prepared and cells were cultured as described previously [28]. Based on the clinical and lung function data, the patients were divided into non-smokers (life-long non-smokers or ex-smokers with less than 10 pack-years), smokers (ex and current smokers) without COPD and smokers with COPD.

### TEM

The patient samples (n = 22) from peripheral lung tissue were analysed by TEM in the cell passages 2–4. The cells were cultured and prepared as described earlier [22,28].

### Western analysis

Cell lines cultured from peripheral lung tissue (n = 22) were analysed by Western analysis for α-SMA as described earlier [28]. The antibodies used are listed in Additional Table 1 in the Additional file 1.

### Statistical analyses

Statistical analyses were performed by Statistical Package for the Social Sciences (IBM SPSS Inc.; version 20.0.0, Chicago, IL) using Chi-Square test or Fishers’ Exact Test for categorized data and Spearman rho Correlation, Mann–Whitney U – test, Independent Samples t-test, Kruskal-Wallis test or ANOVA for continuous data. Values of p < 0.05 were considered as significant and indicated as * < 0.05, ** < 0.01 and *** < 0.001.

## Results

### The alveoli of smokers or COPD displayed less α-SMA positive widened alveolar tips than those of non-smokers

**Figure 1:**
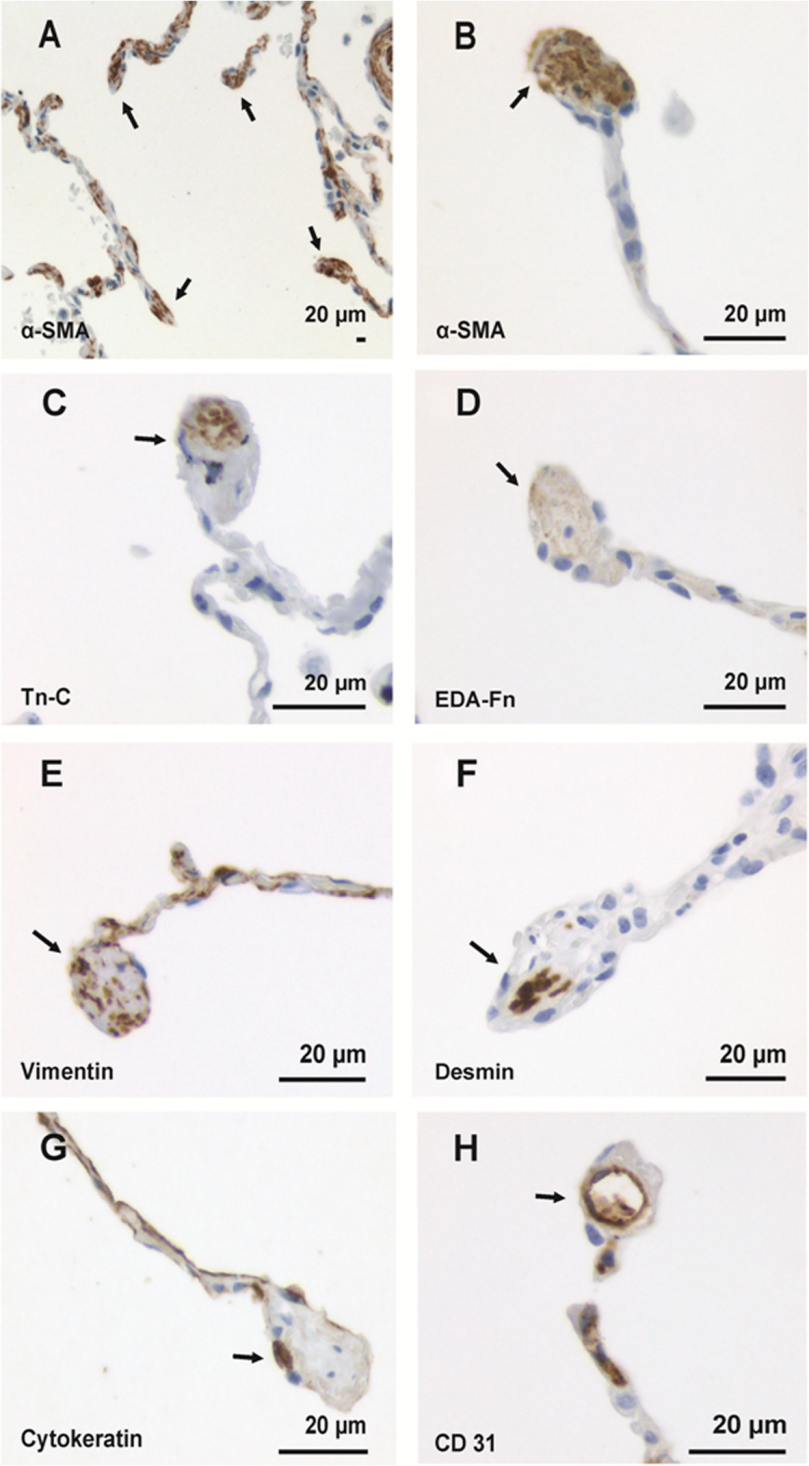
**The immunohistochemical characterization of widened alveolar tips.** Image 1**A** represents alveolar walls and α-SMA positive widened alveolar tips (arrow) from normal peripheral lung tissue in a low power field. Images 1**B-H** show high power fields of a widened tip of a single alveolar septae, which is occupied by α-SMA positive cells (1**B**, arrow), Tn-C (1**C**, arrow), EDA-Fn (1**D**, arrow), vimentin (1**E**, arrow) and desmin (1**F**, arrow). Image 1G illustrates the typical expression of cytokeratin within the edge of a widened alveolar tip (arrow). 1**H** shows a CD31 positive small vessel (arrow) within the widened ending of alveolar septae. Scale bar is shown.

**Figure 2:**
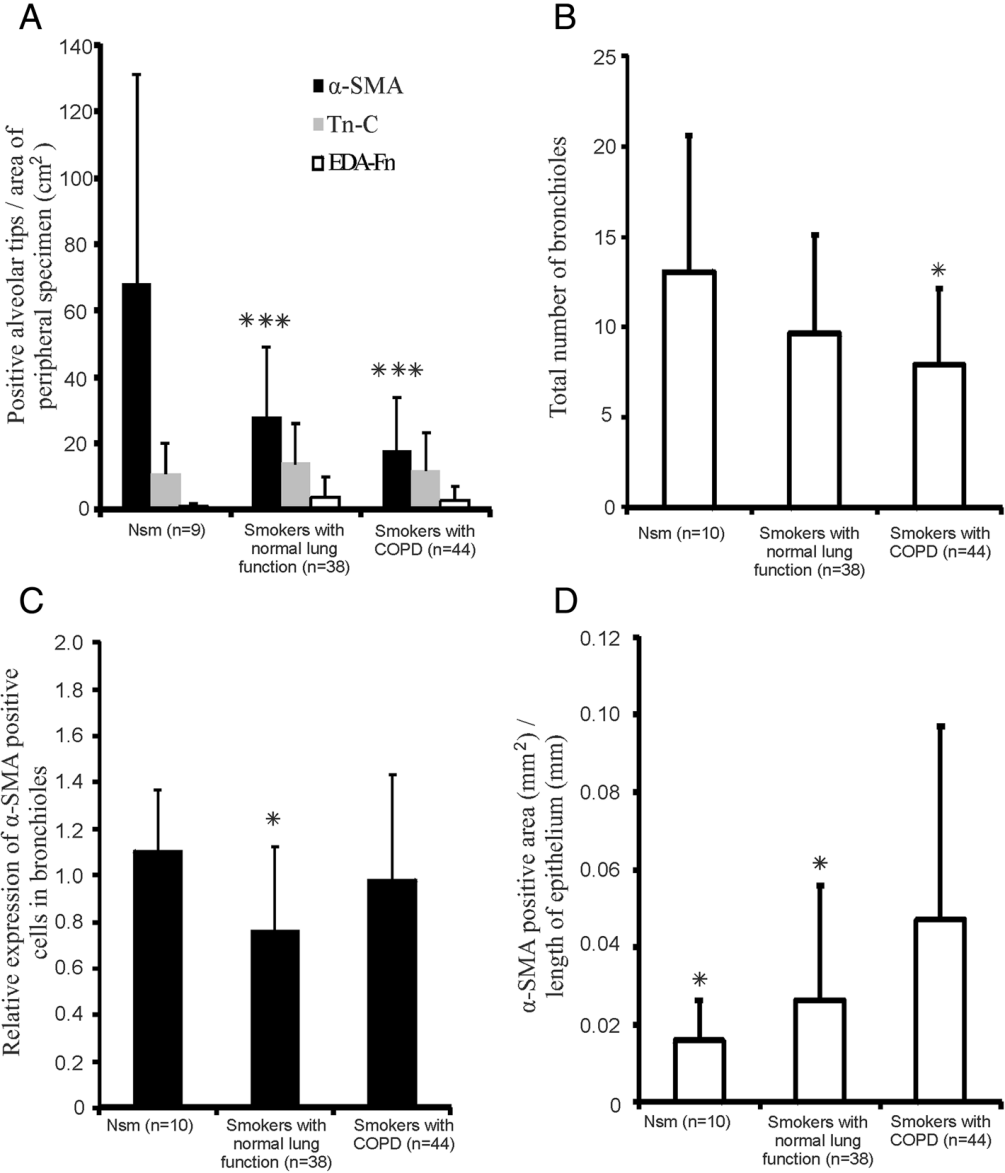
**Number of subjects expressing immunoreactivity for α-SMA, Tn-C or EDA-Fn within widened alveolar tips and airways.** Image 2**A** represents the number of widened alveolar tips, which were positive either for α-SMA (black), Tn-C (grey) or EDA-Fn (white) counted per area of peripheral lung tissue slide (cm^2^) in different patient groups. Stars indicate p < 0.001 when compared to non-smokers. 2**B** shows the total number of small airways i.e. bronchioles in the tissue slide. A star indicates p < 0.05 when compared to non-smokers. 2**C** shows relative expression (weighted mean) of α-SMA positive, spindle-shaped cells in the subepithelial area of bronchioles. A star indicates p < 0.05 when compared to non-smokers. 2**D** illustrates the area of α-SMA positive cells (mm^2^) per length of bronchial epithelium (mm). Stars indicate p < 0.05 when compared to COPD. Standard deviations are shown.

It was observed that many endings of the free interalveolar septa were substantially widened. We named those structures as widened alveolar tips, and observed that they contained α-SMA positive cells, which were obviously myofibroblasts and to a lesser extent also mainly extracellular Tn-C and EDA-Fn positivity, both in normal and diseased lung (Figure 1). Unlike the widened alveolar tips, normal looking alveolar septa without widenings were mostly negative for α-SMA, Tn-C and EDA-Fn. The characteristic features of the widened alveolar tips are shown in Table 2. The smokers with normal lung function (p < 0.001, Dunnett t-test) and the individuals with COPD (p < 0.001, Dunnett t-test) exhibited a reduced number of α-SMA positive widened alveolar tips when compared to the non-smokers. The non-obstructed smokers had a tendency to express more EDA-Fn positive widened alveolar tips than non-smokers (p = 0.059, Dunnett t-test) (Figure 2A). There were no differences detected in the amount of Tn-C expression between any of the groups. There was no difference in the numbers of α-SMA positive widened alveolar tips between various COPD stages.

**Table 2:** Characteristics of immunohistochemical findings in alveoli, bronchioles and bronchi

		**Non-smoker (n = 11)**	**Smoker with normal lung function (n = 41)**	**Smoker with COPD (n = 49)**
**ALVEOLUS**				
*Area of peripheral specimen, cm*^*2*^				
Min		1.4	1.3	1.6
Max		4.8	4.4	4.7
Median		2.8	2.9	2.9
Percentiles	25	2.0	2.4	2.5
	50	2.8	2.9	2.9
	75	3.3	3.3	3.5
α*-SMA in widened tips/cm*^*2*^				
Min		23	6.7	1.4
Max		210	97	77
Median		39	23	14
Percentiles	25	28	11	8.5
	50	39	23	14
	75	98	37	23
*Tn-C in widened tips/cm*^*2*^				
Min		1.5	0	0.7
Max		28	44	44
Median		7.0	8.9	10
Percentiles	25	4.5	5.8	4.2
	50	7.0	8.9	10
	75	17	18	19
*EDA-Fn in widened tips/cm*^*2*^				
Min		0	0	0
Max		2.0	26	13
Median		0.4	1.3	1.8
Percentiles	25	0	0.3	0.7
	50	0.4	1.3	1.8
	75	1.8	7.7	5.6
**BRONCHIOLE**				
*No of bronchioles*				
Min		4.0	1.0	0
Max		30	25	23
Median		11	8.5	7.0
Percentiles	25	9.0	6.0	5.0
	50	11	8.5	7.0
	75	19	14	11
α*-SMA positivity*				
Min		0.8	0	0
Max		1.6	1.6	2.0
Median		1.1	0.8	1.0
Percentiles	25	0.9	0.5	0.6
	50	1.1	0.8	1.0
	75	1.3	1.0	1.4
*Tn-C positivity*		negative	negative	negative
*EDA-Fn positivity*		negative	negative	negative
**BRONCHUS**				
*Epithelium length, mm*				
Min		11	9.7	5.7
Max		62	67	77
Median		30	36	35
Percentiles	25	21	27	28
	50	30	36	35
	75	45	50	46
α*-SMA positive area, mm*^*2*^*/mm*				
Min		0	0	0
Max		0.04	0.10	0.20
Median		0.01	0.02	0.03
Percentiles	25	0.003	0.005	0.01
	50	0.012	0.02	0.03
	75	0.032	0.05	0.07
*Tn-C, no of patients (%)*				
basal cells + BM		9 (90%)	14 (34%)	17 (35%)
basal cells + BM + stroma		0 (0%)	25 (61%)	25 (52%)
basal cells + BM + widely stroma		1 (10%)	2 (5%)	6 (13%)
*EDA-Fn positivity*		negative	negative	negative

α-SMA Alpha-smooth muscle actin, Tn-C Tenascin-C, EDA-Fn Extra type III domain A – fibronectin.

The immunohistochemical stainings of the mesenchymal, epithelial and endothelial markers were analysed in selected cases by using serial tissue sections in order to phenotype the α-SMA positive cells with spindle-shaped morphology within the widened alveolar tips. In serial tissue sections, the spindle-shaped cells in similar locations were positive for both α-SMA and vimentin, which suggested a phenotype typical for myofibroblasts. Most of those cells were negative for desmin since only a few individual cells showed a weak positivity. The widened alveolar tips were lined by cytokeratin positive epithelial cells. We could not detect any CD34, CD45, CD31, PG-M1, E-cadherin, N-cadherin and β-catenin positivity in the spindle-shaped cells within widened alveolar tips (Figure 1). Small vessels positive for CD31 were observed in a few scattered widened alveolar tips.

### The small airways of smokers with normal lung function expressed less α-SMA positive cells than those of non-smokers

**Figure 3:**
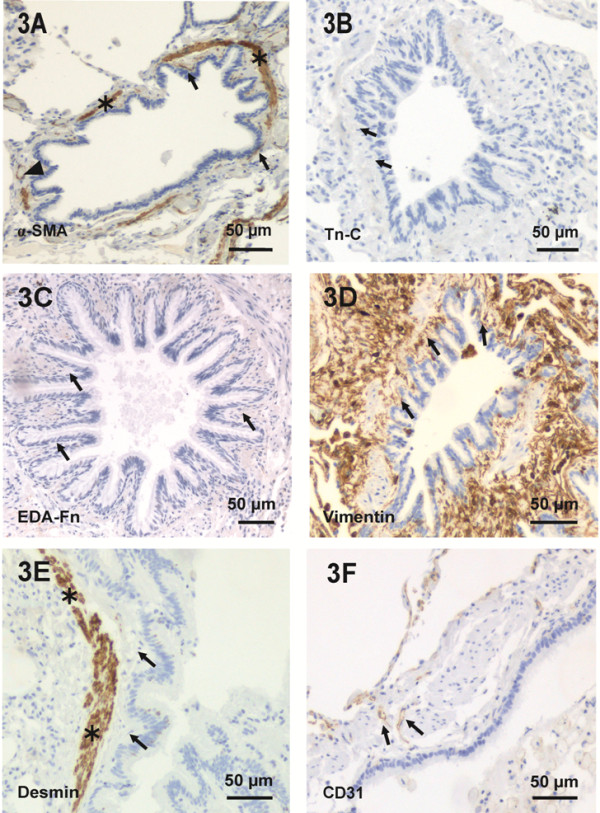
**The immunohistochemical characterization of bronchioles.** Image 3**A** represents a bronchiole with less than 5 α-SMA positive spindle-shaped cells (arrow) within the subepithelial area. The smooth muscle cell layer (asterisk) and small vessels (black arrowhead) were excluded. Images 3**B** and 3**C** reveal that bronchioles were always negative for Tn-C and EDA-Fn (arrows). 3**D** shows a small airway with intensive vimentin positivity (arrows). 3**E** shows desmin positive smooth muscle cells (asterisks) and negative cells (arrows) within the subepithelial area of the bronchiole. 3**F** displays CD31 positive cells (arrows) within the subepithelial area. Scale bar is shown.

The total number of the small airways i.e. bronchioles per tissue slide, was reduced in COPD when compared to that of non-smokers (p = 0.019, Dunnett t-test) (Figure 2B). The bronchioles were either negative for α-SMA or expressed a variable number of α-SMA positive spindle-shaped cells within the subepithelial area of the bronchiolar wall. The immunohistochemical findings are shown in Table 2. The bronchioles of the smokers with normal lung function displayed fewer α-SMA positive spindle-shaped cells than those of the non-smokers (p = 0.016, Mann–Whitney U test). There was no difference between the non-smokers and COPD (Figure 2C). In serial tissue sections, the bronchioles were always negative for Tn-C and EDA-Fn. Vimentin expression was ubiquitously distributed in the subepithelial areas. Spindle-shaped cells in the subepithelial area were negative for desmin whereas only smooth muscle cells showed positive staining. The spindle-shaped cells within the subepithelial area were negative for cytokeratin, N-cadherin, E-cadherin, β-catenin, CD34 and CD45 (Figure 3). There was no difference in the relative expression of α-SMA or in the total number of bronchioles between various COPD stages.

### Numbers of α-SMA positive cells and the extent of Tn-C staining were increased in COPD in the large airways

**Figure 4:**
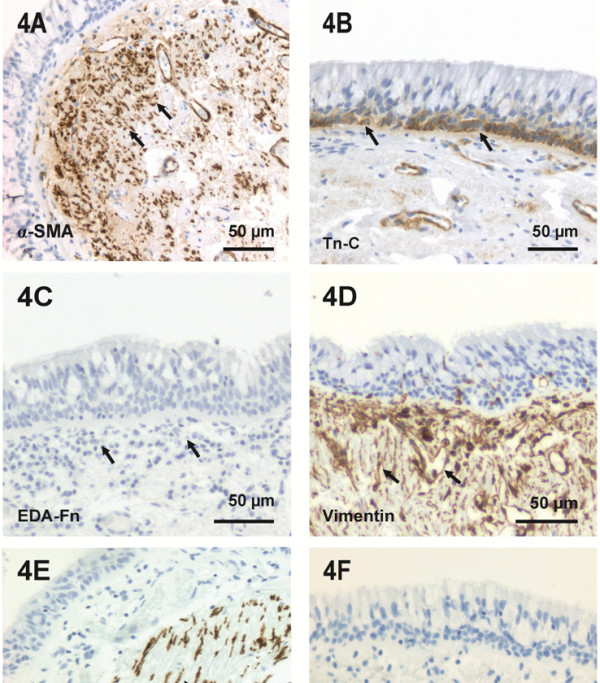
**The immunohistochemical characterization of bronchi.** Image 4**A** represents a group of α-SMA positive spindle-shaped cells (arrows) within the subepithelial area of bronchus. 4**B** illustrates the Tn-C expression typical of normal lung, showing positivity in basal epithelial cells and to a slight extent in basement membrane (arrows). 4**C** represents negative EDA-Fn expression (arrows) in bronchus. 4**D** shows vimentin positive cells in stroma beneath the epithelium (arrows) and a few positive cells among the epithelial cells. 4**E** shows desmin positivity (arrows) within a subepithelial area of bronchus. 4**F** demonstrates mouse isotype control. Scale bar is shown.

The subepithelial area of bronchi expressed α-SMA positive spindle-shaped cells as cell clusters of variable sizes and shapes. The characteristics of the expression of α-SMA in large airways are shown in Table 2. The majority of the spindle-shaped subepithelial cells were positive for α-SMA and vimentin, and mostly negative for desmin. Some cases expressed a few desmin positive cells with spindle-shaped morphology (Figure 4). The expression of α-SMA was elevated in COPD when compared to smokers with normal lung function (p = 0.019, Dunnett t-test) or non-smokers (p = 0.028, Dunnett t-test) (Figure 2D).

All of the cases expressed Tn-C in basal epithelial cells but the level of expression in the subepithelial area of bronchial wall varied. In normal bronchi of non-smokers Tn-C expression was limited to basal epithelial cells and basement membrane (Figure 4). The smokers with normal lung function (p < 0.001, Fisher’s exact test) and COPD (p = 0.002, Fisher’s exact test) expressed more Tn-C beyond the epithelium and basement membrane than non-smokers. The number and proportion of patients within each staining level are shown in Table 2. Ex-smokers with normal lung function had a tendency to express less Tn-C in bronchi (p = 0.056) than current smokers with normal lung function.

The expression for EDA-Fn was negative in all cases (Figure 4). The spindle-shaped cells within the subepithelial areas demonstrated a lack of immunoreactivity for N-cadherin, E-cadherin, β-catenin, CD34 and CD45. There was no difference in expression of α-SMA or Tn-C of bronchi between various COPD stages.

### Pack-years and FEV_1_/FVC correlated with immunohistochemical findings

The correlations were calculated for all patients examined in this study (Table 3). Pack-years correlated negatively with the α-SMA expression in alveoli (p = 0.006, Spearman rho = −0.295) and bronchioles (p = 0.027, Spearman rho = −0.241). In large airways, pack-years correlated positively with Tn-C (p < 0.001, ANOVA). FEV_1_/FVC i.e. obstruction correlated positively with the number of widened alveolar tips that were positive for α-SMA (p < 0.001, Spearman rho = 0.426). FEV_1_ correlated negatively with the number of EDA-Fn positive widened alveolar tips (p = 0.047, Spearman rho = −0.210). DL_CO_ showed a tendency to correlate positively with the α-SMA expression in bronchi (p = 0.055, Spearman rho = 0.234).

**Table 3:** Clinical and immunohistochemical correlations in all patients

		**Clinical parameters**			
Immunohistochemical findings	Sex*	Pack-years	FEV_1_/FVC	FEV_1_	DL_CO_
α-SMA in alveoli	p<0.001	ρ=−0.295 p=0.006	ρ=0.426 p<0.001		
α-SMA in bronchioles		ρ=−0.241 p=0.027			
α-SMA in bronchi	0.05				ρ=0.234 p=0.055
Tn-C in bronchi**	p=0.02	p<0.001			
EDA-Fn in alveoli				ρ=−0.210 p=0.047	

α-SMA Alpha-smooth muscle actin, Tn-C Tenascin-C, EDA-Fn Extra type III domain A – fibronectin, FEV_1_ = Forced expiratory volume in one second, FVC = Forced vital capacity, DL_co_ = Diffusing capacity of carbon monoxide, ρ Spearman’s rho, *study is not sex-matched, ** Correlations of Tn-C were calculated by ANOVA.

Tn-C expression in bronchi correlated with α-SMA in bronchi (p < 0.001, ANOVA) and also with the numbers of widened alveolar tips that were positive for Tn-C (p = 0.01, ANOVA) and EDA-Fn (p = 0.01, Kruskal-Wallis test). The numbers of Tn-C positive widened alveolar tips correlated positively with the numbers of α-SMA (0.016, Spearman rho = 0.254) and EDA-Fn (p < 0.001, Spearman rho = 0.578) positive tips.

When examining the COPD-patients as a separate group, pack-years correlated negatively with the relative α-SMA expression in bronchioles (p = 0.026, Spearman rho = −0.336). FEV_1_/FVC correlated with the number of α-SMA positive widened alveolar tips (p = 0.027, Spearman rho = 0.333). FEV_1_ correlated positively with the α-SMA expression in large airways (p = 0.032, Spearman rho = 0.311). DL_CO_ correlated positively with the total number of bronchioles (p = 0.032, Spearman rho = 0.374), the number of widened alveolar tips positive for α-SMA (p = 0.019, Spearman rho = 0.406), Tn-C (p = 0.009, Spearman rho = 0.448) and EDA-Fn (p = 0.009, Spearman rho = 0.449).

The numbers of widened alveolar tips positive for Tn-C correlated positively with the number of those positive for α-SMA (p = 0.014, Spearman rho = 0.365) and EDA-Fn (p < 0.001, Spearman rho = 0.554). The expressions of α-SMA, Tn-C and EDA-Fn in different lung compartments did not show significant difference between the patients on corticosteroids and patients without the medication.

### In vitro and TEM experiments conducted on cell lines cultured from peripheral lung tissue

The ultrastructural i.e. electron microscopic characterization by TEM revealed that cell populations cultured from peripheral lung tissues were composed of fibroblasts and a small proportion of myofibroblasts. The myofibroblast phenotype was recognized by the ultrastructural characteristics i.e. the expression of a prominent actin belt, extracellular bundle of filaments and dilated rER as described previously [28].

**Figure 5:**
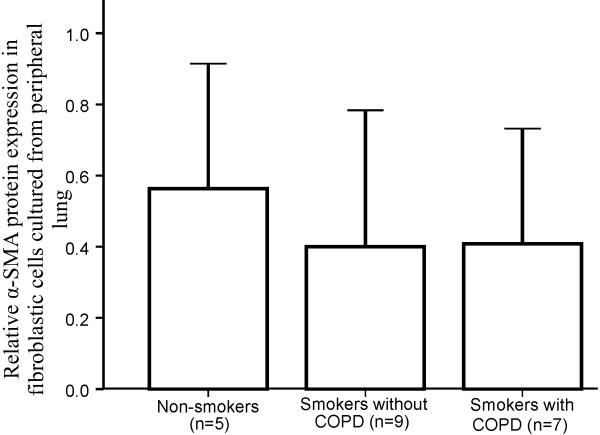
**Cells cultured from peripheral lung of non-smokers, smokers without COPD and COPD were studied by Western blotting for expression of α-SMA protein.** The intensity of α-SMA protein of each sample was referenced to GAPDH and normalized to the control sample. Cells from smokers without COPD and COPD expressed a lower intensity of α-SMA than those derived from normal peripheral lung. Standard deviations are shown.

Western analysis showed that cells isolated from peripheral lung of the smokers without COPD (0.40, SD 0.38) or COPD (0.39, SD 0.31) expressed a 0.70-fold lower amount of α-SMA compared to non-smoking individuals (0.56, SD 0.35) even though the statistical difference was not significant (Figure 5).

## Discussion

The present study demonstrates the lung compartment specific expression of α-SMA positive cells, which are apparently myofibroblasts, in alveoli, bronchioles and bronchi in lung tissue of non-smokers, smokers without COPD and COPD. We observed that the α-SMA positive cells were expressed variably in different areas of lung. Their numbers were decreased in bronchioles and alveoli i.e. in peripheral lung in COPD and smokers when compared to the situation in non-smokers. Cell lines composed of fibroblasts and myofibroblasts *in vitro* cultured from peripheral lung exhibited also a reduced expression of α-SMA in smokers and COPD, which supports the results obtained from the immunohistochemistry. As previously shown, it was also observed that the numbers of small airways were reduced in the lung tissue samples of the patients with COPD [2,29]. The phenomenon of α-SMA expression was different in the large airways since we observed that there were more α-SMA positive cells in COPD and smokers when compared to non-smokers, a finding similar to that described in the previous study [3].

As far as we are aware, this is the first study that has characterized in detail by light microscopy and immunohistochemistry the phenomenon of widenings in free interalveolar septa, which we have termed the widened alveolar tips. It is highly likely that also other investigators have previously detected these structures, although we have not found any previous publication about this topic. Alveolar ducts defined as airway spaces limited to free tips of interalveolar septa have been previously presented but in those studies the widenings of alveolar tips were not described [25,26]. The numbers of α-SMA, Tn-C and ED-A-Fn positive widened alveolar tips were counted in normal and diseased lung, and the immunohistochemical findings were correlated with the clinical data and the smoking history of the patients. Most widened alveolar tips contained spindle shaped cells positive for α-SMA suggestive of a myofibroblast phenotype, and moreover, the numbers of these α-SMA positive alveolar tips were the highest in non-smokers. In addition to α-SMA positivity, a few spindle shaped cells within the tips exhibited positivity for desmin, which indicates that there are two different phenotypes of myofibroblasts, as described previously [30]. The amount of Tn-C or ED-A-Fn positive tips in peripheral lung did not reveal similar clinicopathological correlations as obtained with α-SMA.

During the development of human lung, α-SMA and Tn-C positive structures called secondary crests appear in the walls of the alveoli [19]. The widened alveolar tips described in the present study exhibited a somewhat similar structure as these secondary crests. One could speculate that the alveolar tips are a typical feature of normal alveoli, and may be linked to the regenerative capacity of the adult lung, since alveolar regeneration has been demonstrated to occur also in adult humans [7,31]. Widened alveolar tips may possibly locate at the branching site of alveolar ducts but one would need to examine the exact location of these structures in the lung with three-dimensional methods, which was not possible in the present study. It could be speculated that the widened alveolar tip structure may be caused by an artifact originating during tissue sectioning but against this proposal is the fact that the tips stained positively for several immunohistochemical markers such as α-SMA, Tn-C and EDA-Fn, which were negative in normal looking alveolar walls. On the other hand, it could be possible that the accumulation of positive cells in the widened alveolar tips may be resulted from damaged and weakened alveolar walls.

We observed that smoking and COPD were associated with a lower amount of α-SMA positive widened alveolar tips and also with a lower number of α-SMA positive cells in bronchioles. This finding has to be assessed critically since we did not exclude the emphysematous areas in the measurements of area, which might have effect on the results. Analysing the level of emphysema accurately in histological material is not possible with this kind of retrospective diagnostic lung tissue material.

The widened alveolar tips may be the source of alveolar fibroblasts and myofibroblasts, which may participate in tissue repair and regeneration, whereas long-lasting smoking provokes a decline in the number of myofibroblasts in alveoli and bronchioles thereby contributing to structural changes in COPD, especially in emphysema. Recently it has been shown that the activation of canonical Wnt/β-catenin – signalling pathway precedes myofibroblast transformation in response to TGF-β1 induction in COPD [32], and also that β-catenin was up-regulated during development of human lung [33].

Fibroblasts and myofibroblasts are the main cell types responsible for the synthesis and secretion of the ECM proteins and proteoglycans during tissue repair [14,34]. Annoni and co-workers [5] have recently examined a large spectrum of ECM proteins such as Tn-C, collagens and Fn in normal lung and COPD. They did not analyse the occurrence of α-SMA positive myofibroblasts nor did they evaluate the expression of the factors in the widened alveolar septa. They measured the fractional areas of ECM components based on the color intensity of stainings in subepithelial area, smooth muscle cell layer and outer stroma both in small and large airways. Instead, our image analysis in large airways was based on a categorization of the Tn-C levels by a semi-quantitative method as described previously [3]. Despite the differences in analytical techniques, the result of the present study on Tn-C expression in bronchi was in line with the observation by Annoni and co-workers [5] i.e. Tn-C expression was elevated in COPD.

Annoni and co-workers [5] also observed that the subepithelial area of the small airways in COPD expressed more Tn-C than that of the non-smokers, and normal alveolar walls also expressed Tn-C, the results, which are discrepant with the present and our previous studies. In our previous studies described that Tn-C was scantily expressed in normal human lung [18,22]. One explanation could be that different antibodies for Tn-C have been used in these studies. In humans, Tn-C occurs in nine extra fibronectin type III (FNIII) - repeats due to alternative splicing. This can lead to the assembly of Tn-C subunits with varying numbers and different identities of FNIII repeat [35]. The antibody used in our studies recognized the two major isoforms of Tn-C [36] whereas Annoni with co-workers [5] used the antibody that recognized all Tn-C isoforms.

The present study showed that the cells cultured from peripheral lung of non-smokers displayed a tendency to express more α-SMA protein by Western analysis than the cells derived from smokers with normal lung function or COPD. Even though there was no statistical significant difference between any of the groups, this finding is in line with the immunohistochemical results. Other studies have reported that the fibroblasts cultured from distal lung from individuals with COPD exhibited a trend toward increased α-SMA expression [37,38]. This discrepancy with our findings and others may be explained by the different severities of COPD stages in these abovementioned studies. Togo with co-workers [37] studied cells from the patients with moderate to very severe COPD stages (stage II-IV) and Hallgren with co-workers [38] from patients with very severe COPD (stage IV), whereas our study mostly included patients with mild or moderate COPD (stage I-II). It is possible that the amount of α-SMA positive cells decreases in mild COPD, but increases in severe COPD, a phenomenon which is supported by the similar findings from a previous study analysing precursor proteins of collagens in COPD [2].

## Conclusions

We conclude that the role of α-SMA positive cells, which are apparently myofibroblasts, in alveoli and bronchioles might be involved in the regeneration of the adult lungs as they are expressed in the pulmonary tissue of non-smokers, healthy smokers and COPD. On the other hand, in large airways the numbers of these cells are increased in COPD potentially participating in the pathogenesis of the disease. The newly described structure, which was termed the widened alveolar tip, is the source of the obvious myofibroblasts and ECM proteins at the alveolar level. The widened alveolar tips are expressed both in normal and diseased lung. Further investigations are needed to elucidate whether the widened alveolar tips take part in the alveolar regeneration in the adult lungs.

## Abbreviations

COPD: Chronic obstructive pulmonary disease; α-SMA: Alpha-smooth muscle actin; Tn-C: Tenascin-C; ECM: Extracellular matrix; Fn: Fibronectin; IPF: Idiopathic pulmonary fibrosis; RDS: Respiratory distress syndrome; BPD: Bronchopulmonary dysplasia; EDA-Fn: Extra type III domain A – fibronectin; TGF-β1: Transforming growth factor – beta 1; rER: Rough endoplasmic reticulum; TEM: Transmission electron microscopy; GOLD: Global initiative for chronic obstructive pulmonary disease; BM: Basement membrane; FNIII: Fibronectin type III; FEV1: Forced expiratory volume in one second; FVC: Forced vital capacity; FEV1/FVC: The ratio of forced expiratory volume in one second to forced vital capacity; DLCO: Diffusing capacity of carbon monoxide; Postbd: Post-bronchodilator value.

## Competing interests

The authors report no competing interest.

## Authors’ contributions

HMK analysed immunohistochemistry and image analysis, performed cell culture and cell experiments, participated in the data analyses, and prepared the draft of the manuscript. STL collected cell culture material and participated in cell culture experiments, image analyses and Western analysis. TH analysed clinical data of patients. RTS was responsible for electron microscopic interpretations. ELB evaluated histological material and collected material for cell culture experiments. JMM evaluated histological material. KL and SJ collected the clinical data of the patients. RLK designed the study, collected and analysed clinical and histological data, planned and analysed immunohistochemistry. All authors participated in manuscript preparation. All authors read and approved the final manuscript.

## Supplementary Material

Additional file 1 Additional information:
Myofibroblast expression in airways and alveoli is affected by smoking and COPD. Click here for file

Additional file 2: Figure S1 More information about the image analyses of α-SMA and Tn-C in bronchioles and bronchi. Click here for file
