# Supplementary material for: Myofibroblast expression in airways and alveoli is affected by smoking and COPD
Source: Respir Res. 2013 Aug 11;14(1):84. doi: 10.1186/1465-9921-14-84 (PMC3751100; doi:10.1186/1465-9921-14-84)
Supplement: Additional file 1 — Additional information: Myofibroblast expression in airways and alveoli is affected by smoking and COPD. [file 1465-9921-14-84-S1.pdf]

Additional Information

Myofibroblast expression in airways and alveoli is affected by smoking and COPD

Henna M Karvonen[1,2], Siri T Lehtonen[2,3], Terttu Harju[1,2], Raija T Sormunen[4,5,6], Elisa Lappi-Blanco[5,6], Johanna M Mäkinen[5,6], Kirsi Laitakari[1,2], Shirley Johnson[1,2] and Riitta L Kaarteenaho[2,7]

Authors' affiliations

[1] Department of Internal Medicine / Respiratory Research Unit, Institute of Clinical Medicine, University of Oulu, Oulu, Finland

[2] Respiratory Research Unit and Medical Research Center Oulu, Oulu University Hospital, Oulu, Finland

[3] Department of Anatomy and Cell Biology, Institute of Biomedicine, University of Oulu, Oulu, Finland

[4] Biocenter Oulu, University of Oulu, Oulu, Finland

[5] Department of Pathology, Institute of Diagnostics, University of Oulu, Oulu, Finland

[6] Department of Pathology, Oulu University Hospital, Oulu, Finland

[7]Unit of Medicine and Clinical Research, Pulmonary Division, University of Eastern Finland and Center for Medicine and Clinical Research, Division of Respiratory Medicine, Kuopio University Hospital, Kuopio, Finland

Methods

Ethical considerations

The study had the approval from the Ethical Committee of Northern Ostrobothnia Hospital District in Oulu (statements 64/2001, amendment 68/2005, 2/2008), and from National Supervisory Authority for Welfare and Health (former National Authority of Medicolegal Affairs, reg. nr. 7323/05.01.00.06/2009 and 863/04/047/08). For the retrospective immunohistochemical material, informed consent permission has been given by the National Supervisory Authority for Welfare and Health, which is the national licensing authority. The study material for experiments conducted on cell lines was collected prospectively, when the patients were interviewed before the operation and samples were collected only if written consent was given.

Immunohistochemistry

Formalin-fixed and paraffin-embedded specimens (1-2 cm^2) were cut into 4.5 µm sections, de-paraffinized in xylene and rehydrated in a descending ethanol series. Endogenous peroxidase was inactivated by Peroxidase-Blocking Solution (Envision+ System kit, Dako). The stainings were performed as described previously [1] using Envision+ System Kit (Dako, Denmark) with DAB 3,3' diaminobenzidine chromogen by Dako Autostainer plus (Dako) staining equipment. The samples were cooked by Tris-EDTA (pH 6) for 15 min or by citrate buffer for 10 min (indicated in Additional Table 1). Phosphate-buffered saline (PBS) and isotype control were used as negative controls. For selected cases (5 samples from peripheral

lung and 5 samples from bronchi) were studied in detail for the markers of epithelial, endothelial and mesenchymal origin. The specimens were visualized by light microscope (Leica DM 3000 equipped with ∞/-/B N Plan objective, a digital camera Leica DFC420 and software Leica Application Suite LAS version 3.1.0.).

Antibodies

Antibodies used in immunohistochemistry and Western analysis are shown in Additional Table 1.

In vitro experiments conducted on cell lines cultured from peripheral lung tissue

The peripheral lung tissues from lung resections were prepared and cells were cultured as described previously [2]. Tissue samples were washed with ice-cold PBS, cut into approximately 1 mm^3 pieces and incubated with collagenase I (500 U/ml) - DNAase I (20 U/ml) at +37° C / 5 % CO_2 for 3 h. The pieces were placed in culture medium (Minimum essential medium Eagle, α-modification (Sigma, Steinheim, Germany) containing 13 % fetal bovine serum (FBS-500, PromoCell, Heidelberg, Germany), 10 mM 4-(2-hydroxyethyl)-1-piperazine-ethanesulfonic acid (HEPES, Sigma), 100 U/ml penicillin (Sigma), 100 µg/ml streptomycin (10 mg/ml, Sigma), 2.5 µg/ml amphotericin B (Sigma) and 2 mM L-glutamine (Sigma)) and allowed to attach for one day. The remaining tissue pieces were washed away. Cells were let to grow up to 80 % of confluence, passaged (5 mg/ml trypsin - 2 mg/ml EDTA

(Sigma)) and plated at a density of 2 500 cells per cm^2. Cells in passages 2-4 were used in the experiments.

TEM

The specimens were prepared as previously described [1-2]. The samples from peripheral lung of every patient were analysed. Cells were fixed in 1 % glutaraldehyde and 4 % PFA mixture in 0.01 M phosphate buffer (PBS) for 10 min. The cells were scraped off, pelleted and fixation was continued for 1 h. The pellet was immersed in 2 % Agarose (in dH_2O) and postfixed in 1 % osmium tetroxide in dH_2O for 30 min. The specimens were dehydrated in acetone and immersed in Epon LX112 (Ladd Research Industries, Vermont, USA)-acetone. Thin sections (80 nm) were cut with Leica Ultracut UCT ultramicrotome (Leica Microsystems, Vienna, Austria) and stained with uranyl acetate and lead citrate and examined in a Philips CM 100 transmission electron microscope (FEI company, Eindhoven, The Netherlands) equipped with a Morada CCD camera (Olympus Soft Imaging System Solutions GMBH, Münster, Germany).

Western analysis

The blotting was done as previously described [2]. Cells were lysed in lysis buffer (50 mM Tris pH 7.6, 0.1 % Triton X-100, 0.9 % NaCl, 0.2 % sodium azide (Sigma), 0.1 % deoxycholic acid (DOC, Sigma), 1x Protease Inhibitor Cocktail Tablet (Roche, Mannheim,

Germany)), incubated on ice for 30 min and centrifuged (10 000 rpm, 20 min, +4 °C). The protein concentration of samples was determined by the microplate assay (DC Protein Assay Kit, Bio Rad, UK) according to the manufacturer's instructions. In brief, 20-µg aliquots in sample buffer (0.1 M Tris-HCl pH 6.8, 0.2 M dithiothreitol, 25 % glycerol, 1mg/ml bromophenol blue, 4 % SDS) were loaded on 12 % SDS-PAGE with running buffer (25 mM Tris, 190 mM glycine, 0.1 % SDS) at 200 V. After the electrophoresis, the proteins were transferred to nitrocellulose membrane (pore size 0.45 µm, Protran® Nitrocellulose Transfer Membrane, Schleicher & Schuell, BioScience, Dassel, Germany) with blotting buffer (30 mM Tris, 0.24 M glycine, 20 % ethanol) overnight and blocked in 5 % milk powder-PBST (PBS-0.1 % Tween 20) for 1 h. After washing with PBST, the membrane was incubated with the primary antibody against human α-SMA and reference protein GAPDH followed by the secondary antibody (Additional Table 1). The intensity of protein was analysed with an Odyssey infrared imager (LI-COR Biosciences). Equal loading and transfer were confirmed by staining some membranes with 0.1 % Ponceau S in 5 % acetic acid or by glyceraldehyde-3-phosphate dehydrogenase (GAPDH) detection. The data were reported as relative protein expression such that intensities detected for each sample were normalized against the intensity of the control sample on the same membrane. The same control sample was used on every gel/membrane to minimize the variation between different membranes. Cells derived from a patient with normal peripheral lung were used as a control sample.

References

1. Kaarteenaho-Wiik, R, Pääkkö, P, Sormunen, R: **Ultrastructural features of lung fibroblast differentiation into myofibroblasts.** *Ultrastruct Pathol* 2009, **33**(1): 6-15.

2. Karvonen, HM, Lehtonen, ST, Sormunen, RT, Harju, TH, Lappi-Blanco, E, Bloigu, RS, Kaarteenaho, RL: **Myofibroblasts in interstitial lung diseases show diverse electron microscopic and invasive features.** *Lab Invest* 2012, **92**(9)**:** 1270-1284.

Endnotes

Endnote to Additional Table 1

*Tris-EDTA cooking for 15 min, **Citrate buffer cooking for 10 min, [#] EDA-Fn expression was confirmed by stronger concentration (1:500), WB = Western analysis, RT = room temperature, o / n = overnight incubation

Figure legends

Additional Table 1. Antibodies used in immunohistochemistry and Western analysis

Additional Figure 1. Immunohistochemistry and image analyses of bronchioles and bronchi. Smooth muscle cells (asterisk) and small vessels (arrow head) were not counted. Figures A-C show representative micrographs of bronchioles from each category graded as negative (A), 1

(B) or 2 (C) based on the number of α-SMA positive spindle-shaped cells (black arrow) within subepithelial area of bronchiolar wall (marked as red line). Figure D shows an example image of bronchus, which is negative for α-SMA. Figure E demonstrates the area of α-SMA positive spindle-shaped cells within the subepithelial area of bronchus (red line). Figures F-H represent high power fields of bronchi, which show Tn-C positivity (black arrow) in basal epithelial cells and along the BM of bronchial epithelium (F), in basal cells plus BM plus to some extent in the stroma underneath BM (G), and in the above-mentioned areas plus extensively in the stroma (H). Scale bar is shown. BM = basement membrane

Tables

Antibody	Clone	Manufacturer	Source	Dilution	Incubation
monoclonal anti-human α-SMA	1A4	Dako, Glostrup, Denmark	mouse	1:500<br>1:1000 (WB)	30 min at RT*<br>o/n at +4° C (WB)
monoclonal anti-human tenascin-C	DB7	BioHit, Helsinki, Finland	mouse	1:7000	1h at RT*
monoclonal anti-human EDA-fibronectin	IST-9	Abcam, Cam-bridge, UK	mouse	1:1000[#]	1h at RT*
monoclonal anti-vimentin	V9	Dako, Glostrup, Denmark	mouse	1:1500	30 min **
monoclonal anti-human desmin	D33	Dako, Glostrup, Denmark		1:300	30 min*
monoclonal anti-human cytokeratin	AE1/AE3	Dako, Glostrup, Denmark	mouse	1:700	30 min*

monoclonal anti-human CD31	JC70A	Dako, Glostrup, Denmark	mouse	1:400	30 min*
monoclonal anti-human CD68	PG-M1	Dako, Glostrup, Denmark	mouse	1:100	30 min*
monoclonal anti-human CD45	2B11 + PD7/26	Dako, Glostrup, Denmark	mouse	1:400	30 min*
monoclonal anti-human CD34	OBEnd/10	Novocastra, Hämeenlinna, Finland	mouse	1:500	30 min*
monoclonal anti-human β-catenin	14	BD Biosciences, NJ, USA	mouse	1:800	60 min**
monoclonal anti-human E-cadherin	HECD-1	Zymed Laboratories Inc, San Francisco, USA	mouse	1:300	30 min*
monoclonal anti-human N-cadherin	3B9	Zymed Laboratories Inc, San Francisco, USA	mouse	1:100	30 min*

polyclonal anti-human GAPDH Antibody	Abcam, Cambridge, UK	rabbit	1:2500	o / n at +4 °C
Secondary anti-mouse IgG	Zymed Invitrogen, Carlsbad, USA	rabbit	1:1000	30 min at RT
IRDye 800 Conjugated Affinity Purified anti-mouse IgG (H&L)	Rockland Immunochemicals, Inc., Gilbertsville, PA, USA	donkey	1:1000	1 h at RT
IRDye 700DX Conjugated Affinity Purified anti-rabbit IgG (H&L)	Rockland Immunochemicals, Inc., Gilbertsville, PA, USA	donkey	1:1000	1 h at RT
