# Supplementary figures and images for: Myofibroblast expression in airways and alveoli is affected by smoking and COPD
Source: Respir Res. 2013 Aug 11;14(1):84. doi: 10.1186/1465-9921-14-84 (PMC3751100; doi:10.1186/1465-9921-14-84)

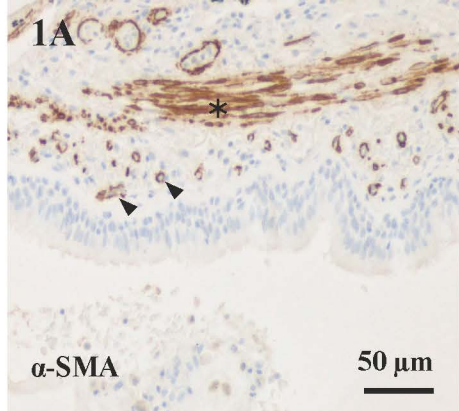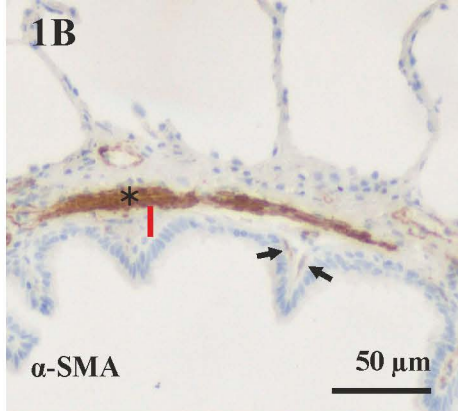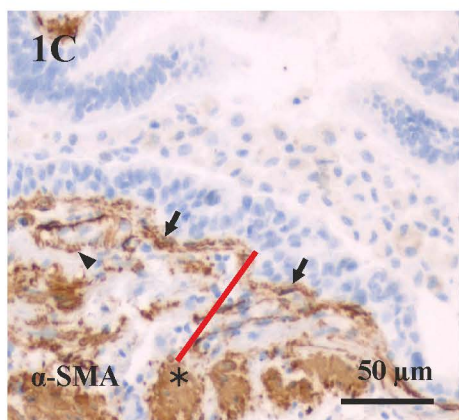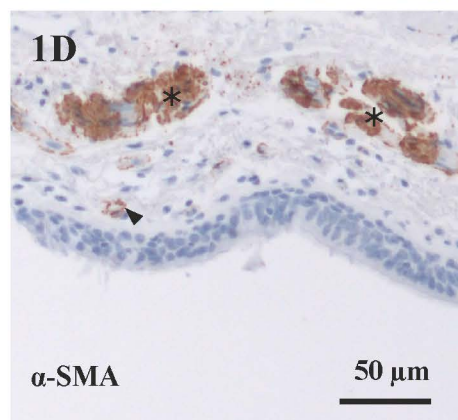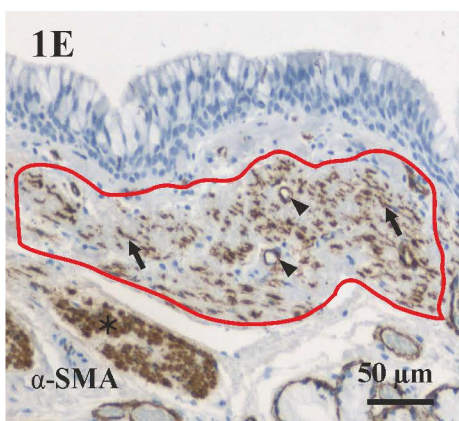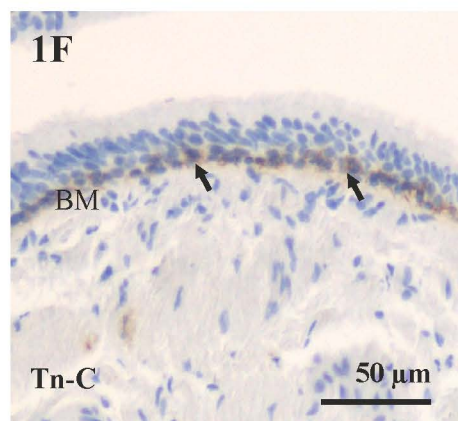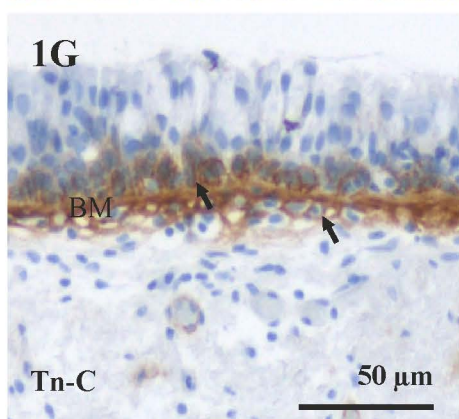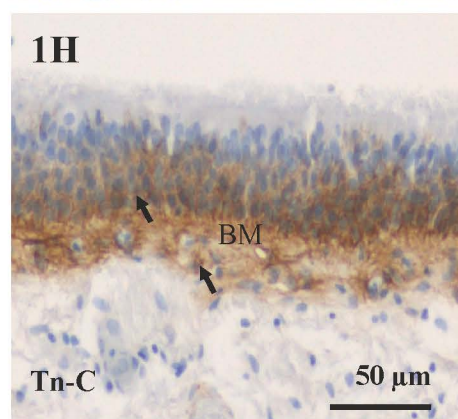

Supplement: Additional file 2: Figure S1 — More information about the image analyses of α-SMA and Tn-C in bronchioles and bronchi. [file 1465-9921-14-84-S2.pdf]
